# Botulinum Toxin Versus Placebo: A Meta-Analysis of Treatment and Quality-of-life Outcomes for Hyperhidrosis

**DOI:** 10.1007/s00266-021-02140-7

**Published:** 2021-02-22

**Authors:** Doha Obed, Mustafa Salim, Alperen S. Bingoel, Thurid R. Hofmann, Peter M. Vogt, Nicco Krezdorn

**Affiliations:** 1 Department of Plastic, Aesthetic, Hand and Reconstructive Surgery, Hannover Medical School, Carl-Neuberg-Strasse 1, 30625 Hannover, Germany; 2 Department of Hematology and Oncology, University Medical Center Goettingen, Goettingen, Germany

**Keywords:** Botulinum toxin, Botox, Hyperhidrosis, Randomized controlled trial, Meta-analysis

## Abstract

**Aims:**

This study aims at assessing the treatment effect, disease severity and quality-of-life outcomes of botulinum toxin (BTX) injections for focal hyperhidrosis.

**Methods:**

We included randomized controlled trials of BTX injections compared with placebo for patients with primary or secondary focal hyperhidrosis. PubMed, Embase and the Cochrane Library were searched to August 2020. Gravimetric sweat rate reduction, disease severity measured by Hyperhidrosis Disease Severity Scale and quality-of-life assessment measured by Dermatology Life Quality Index were the outcomes of interest. Cochrane risk-of-bias tools were employed for quality assessment of given randomized controlled trials.

**Results:**

Eight studies met our inclusion criteria (*n*=937). Overall, risk bias was mixed and mostly moderate. BTX injections showed reduced risk in comparison with placebo for the gravimetric quantitative sweat reduction of > 50 % from baseline (risk difference: 0.63, 95% CI 0.51 to 0.74). Additionally, improvements were seen for disease severity and quality-of-life assessments evaluated by Hyperhidrosis Disease Severity Score reduction of ≥ 2 points (risk difference: 0.56, 95% CI 0.42 to 0.69) and mean change in Dermatology Life Quality Index (mean difference: − 5.55, 95% CI − 7.11 to − 3.98). The acquired data were insufficient to assess for long-term outcomes and limited to an eight-week follow-up period.

**Conclusions:**

In focal axillary hyperhidrosis, BTX significantly reduces sweat production and yields superior outcomes in assessments of disease severity and quality-of-life. However, the quality-of-evidence is overall moderate and included studies account for short-term trial periods only. Further studies assessing BTX in comparison with first-line treatments for hyperhidrosis are warranted.

**Level of Evidence III:**

This journal requires that authors assign a level of evidence to each article. For a full description of these evidence-based medicine ratings, please refer to the Table of Contents or the online Instructions to Authors www.springer.com/00266.

## Introduction

Hyperhidrosis is a chronic pathological disorder that is marked by excessive sweating beyond that which is required to maintain the body’s physiologic homeostatic thermoregulation. The underlying mechanism is based on the overstimulation of cholinergic receptors of eccrine glands, which are highly concentrated in areas such as the palms, soles, axillae and face [1]. With an estimated prevalence of approximately 3%, idiopathic primary hyperhidrosis per definition shows a bilateral and symmetric pattern in one or several sites of predilection, occurs more than once weekly with consequent disruptions of daily activities, is absent nocturnally, lasts at least for 6 months and typically manifests during puberty and adolescence [2]. In contrast, secondary hyperhidrosis is linked to underlying medical conditions, including endocrine or metabolic disorders and malignancies, and the intake of systemic medications such as psychotropic agents or steroids. In addition to the confirmation of diagnosis derived by clinical evaluation, it can be supported by quantitative sweat production analysis (gravimetry) and iodine starch testing [3]. Hyperhidrosis can significantly impair patients’ psychosocial quality of life and be a long-term source of detrimental emotional and physical distress.

Several different therapeutic modalities, surgical and non-surgical, have been established, ranging from topical medications, systemic agents, iontophoresis and endoscopic sympathectomy, with a variable response to available regimens depending on the site of treatment [4]. Similarly, radiofrequency thermotherapy and fractional microneedle radiofrequency have emerged in recent years as alternative treatment modalities. Attempts to reduce the severity and impairment of quality-of-life by hyperhidrosis with given treatment options have had mixed results. First-line non-surgical options such as topical antiperspirants, e.g., aluminum chloride, often tend to alleviate symptoms only short-term, lack effectiveness in sweat reduction, necessitate regular reapplication and often cause secondary dermal skin conditions [5]. Surgical measures, such as endoscopic sympathectomies or sweat gland excisions, are comparatively invasive and bear a high risk for major complications such as wound healing impairment and infections, and often require additional resources such as anesthesia. Accordingly, the optimal treatment agent resulting in ameliorated patient satisfaction would be non-surgical and effective in the long run without the downsides of dermal complications [6].

The injection of the neurotoxin botulinum toxin (BTX) has gained popularity as a treatment option, owing to its high anhidrotic efficacy. By irreversibly inhibiting acetylcholine release from presynaptic vesicles, the minimally invasive procedure allows for the block of cholinergic pathways to apocrine and eccrine glands and thereby results in considerable reduction of perspiration [7].

First-line topical and oral regimens are widely used for the treatment of hyperhidrosis; however, their use is frequently met by unsatisfactory results and adverse events such as skin irritation or dryness of surrounding tissues [8]. Several randomized clinical trials have investigated the use of BTX injections in patients with hyperhidrosis in comparison with placebo and alternative treatment modalities. Mostly, the use of the neurotoxin has proven to be clinically effective and successful in increasing patient satisfaction when employed [9].

Study features such as treatment regimen, therapeutic site, duration of application, patient characteristics, severity of disease and sample size can differ between trials, resulting in a compromised generalizability of given individual studies. Accordingly, this meta-analysis aimed to examine the efficacy of BTX injections versus placebo in patients over the age of 16 years suffering from hyperhidrosis.

## Methods

### Search Strategy

A thorough systematic and comprehensive literature search was conducted without language or date restrictions within databases including PubMed, Embase and the Cochrane Library up until August 2020. MESH search terms and a combination of index terms used included “hyperhidrosis,” “sweat,” “botulinum toxin” and “randomized controlled trial” and comprised all term variations. Citations given in all included studies were manually examined and reviewed. Moreover, the leading European, American and Asian BTX manufacturers were contacted for additional and non-listed articles.

### Selection Criteria

We included all randomized controlled trials that reported on the efficacy and safety of BTX injections (regardless of serotype) in patients suffering from primary and secondary focal hyperhidrosis of any region over the age of 16 years. Eligible studies had to compare BTX injections with placebo injections. Studies that did not include placebo treatment and that were performed in younger age groups or healthy individuals were excluded.

### Outcome Measures

The primary outcome was a gravimetric sweat reduction of ≥ 50% from baseline at week 2–6, as an objective parameter to determine treatment efficacy. Since hyperhidrosis impairs the quality-of-life, various assessment questionnaires are used to depict that negative impact. The Hyperhidrosis Disease Severity Scale (HDSS) scores the tolerability of sweating and its effect on the patient’s life on a 4-point scale, ranging from non-noticeable sweating up to unbearable and daily-life impairing sweating. Similarly, the Dermatology Life Quality Index is also frequently assessed to evaluate the severity of the condition. Therefore, secondary outcomes were the reduction of ≥ 2 points on the Hyperhidrosis Disease Severity Scale within 2–8 weeks and the mean reduction of the Dermatology Life Quality Index score from baseline within 2–8 weeks.

### Literature Screening

Upon the database search in the aforementioned databases, search results were imported into Endnote X7 software. Duplicates were identified and subsequently deleted. After predeterminations of selection criteria, the literature search and evaluation were conducted by two researchers independently (DO and MS). After drawing a first selection of studies based on abstracts and titles, we followed up with a second evaluation after obtaining the full texts to check eligibility for the final analysis. Discrepancies during the screening process were discussed by the two reviewers after each stage in order to reach consensus.

### Data Extraction

Data extraction was performed independently by two researchers (DO and MS) and comprised: the first author, publication year, characteristics of the trial, study period, number of total and subgroup patients enrolled, demographics (age, sex, country), localization of hyperhidrosis, treatment regimen and dosage, BTX manufacturer, treatment duration and discontinuation and the study results. Again, conflicting data extractions were discussed and adjusted upon agreement.

### Assessment of Quality

All of the included studies were randomized controlled trials, and their quality was independently weighed by two reviewers (DO and MS) employing the Cochrane Collaboration’s tool [10], which allows for the evaluation of risk bias (low, high or unclear) of six particular domains comprising randomization, blinding of subjects and outcome assessors, possible sources of bias, allocation concealment, reporting of incomplete outcome data or selective outcome reporting.

### Statistical Analysis

We performed a meta-analysis of the data from a total of eight included studies using RevMan 5.3 software (Review Manager (RevMan), Version 5.3. Copenhagen: The Nordic Cochrane Centre, The Cochrane Collaboration 2014). To evaluate the risk difference in dichotomous outcomes and the mean difference in continuous outcomes with 95% confidence intervals in forest plots, the random effects model through the Mantel–Haenszel estimator was chosen due to the various populations’ origins. The studies’ heterogeneity was evaluated with the *I*^2^ statistic and the Chi-squared test. *I*^2^ values *>* 50% implied significant heterogeneity. Additional subgroup analyses were conducted to identify potential sources of heterogeneity. Meta-regressions and further subgroup analyses were not considered due to the small number of included studies.

## Results

### Literature Selection and Basic Information

**Fig. 1:**
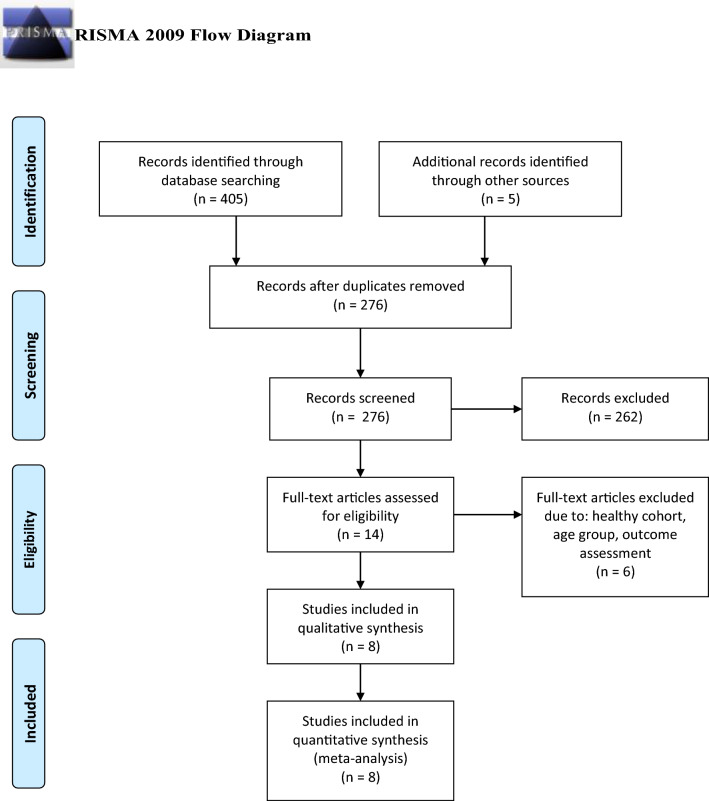
PRISMA flow diagram of the study selection process

The literature screening process is portrayed in the PRISMA flowchart [11] (Fig. 1). A total of 410 articles were identified upon initial literature search. A total of 134 duplicate entries were recognized and eliminated, resulting in 276 studies. From these, 262 articles were excluded after title and abstract evaluation and six articles were excluded after full-text screening based on given exclusion criteria. In total, eight studies were included in our meta-analysis for final evaluation [12–19]. A total of 937 patients were comprised in the included studies. All subjects suffered from focal hyperhidrosis affecting the axillae, the craniofacial or the lower limb areas. The treatment modalities employed in the included studies were BTX-A injections in six trials and BTX-B injections in two trials. All analyses were conducted in comparison with placebo injections. Table 1 summarizes the details of the eight eligible studies, respectively.

**Table 1:** Overview of the study characteristics

First author	Cabreus	Connor	Heckmann	Lowe	Naumann	Odderson	Ohshima	Pasquina
Year	2019	2006	2001	2007	2001	2002	2013	2016
Study design	RTC	RTC	RTC (half-sided)	RTC	RTC	RTC	RTC	RTC (half-sided)
Country	Sweden	USA	Germany	USA	Belgium, Germany, Switzerland, UK	USA	Japan	USA
Age group	17–84	18–65	N/A	18–69	18–75	16–50	N/A	21–33
No. patients	8	33	145	252	320	18	152	9 (11 lower limb amputations)
No. patients BTX	3	15	145	179	234	12	78	7 (limbs)
No. patients control	5	18	145	73	73	6	74	4 (limbs)
Study period	4 weeks	8 weeks	15 months	52 weeks	16 weeks	5 months	24 weeks	37 months
Hyperhidrosis	primary	secondary	primary	primary	primary	primary	primary	secondary
Focal site	Cranio-facial	Axillae (bilateral)	Axillae (bilateral)	Axillae (bilateral)	Axillae (bilateral)	Axillae (bilateral)	Axillae (bilateral)	lower limbs (transfemoral n=4; transtibial n=7)
BTX serotype	BTX B (rima-botulinum-toxin B)	BTX A (ona-botulinum-toxin A)	BTX A (abo-botulinum-toxin A)	BTX A (ona-botulinum-toxin A)	BTX A (ona-botulinum-toxin A)	BTX A (ona-botulinum-toxin A)	BTX A	BTX B (rima-botulinum-toxin B)
BTX manufacturer	Eisai Europe	Allergan	Ipsen Pharma	Allergan	Allergan	Allergan	N/A	Elan Pharmaceuticals
BTX dosage	2.250 U (250 U/mL) total	50 U per Axilla	200 U per Axilla (vial: 500 U BTX, 0.125 mg human albumin, 2.5 mg lactose in 5 ml 0.9 % sodium chloride solution)	50 or 75U per Axilla	50 U per Axilla	50 U per Axilla	50 U per Axilla	2500 U/ml (4 ml per residual transtibial limb, 8 ml per residual transfemoral limb)
Control medication/Placebo	Placebo	Placebo	Placebo	Placebo	Placebo	Placebo	Placebo	Placebo
Control dosage	0.9% sodium chloride (9 ml)	0.9% sodium chloride (4 ml)	0.125 mg human albumin and 2.5 mg lactose (in 5 ml 0.9% sodium chloride solution)	0.9% sodium chloride (2 ml)	0.9% sodium chloride (2 ml)	0.9% sodium chloride (1 ml)	0.9% sodium chloride (2 ml)	0.9% sodium chloride (4 ml transtibial, 8 ml transfemoral)
Follow-up	Week 3 ± 1	Week 8	Week 2, 4, 12, 24, 14, 26	Week 1, 4, 8 and every 4 weeks	Week 1, 4, 8, 12, 16	Month 1, 2, 3, 4 and 5	Week 4, 8, 12, 16	Week 4-6
Commercial sponsors	No	Yes	No	Yes	Yes	No	No	No

### Quality Assessment

**Fig. 2:**
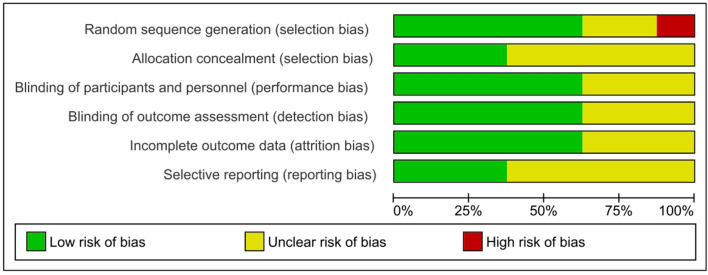
Risk of bias graph presented as percentages across all included studies

**Fig. 3:**
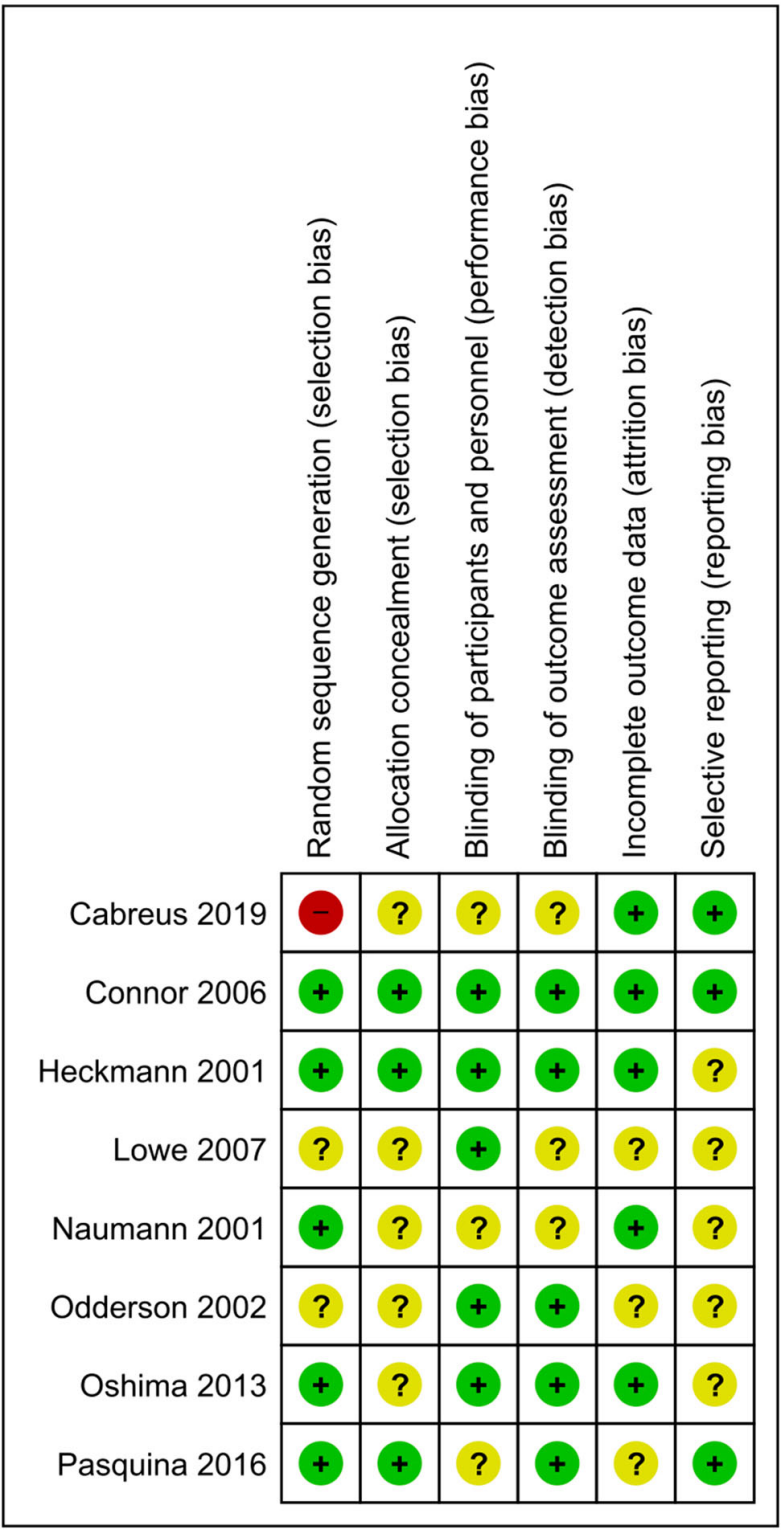
Risk of bias summary with detailed assessment of all included studies

All studies were assessed with the help of Cochrane’s Collaboration’s tool. Allocation concealment was only described in three of the included studies [12, 14, 19]. Other domain assessment revealed a significant bias regarding allocation concealment and selective reporting. Most studies did not allow for complete assessment of reporting bias due to insufficient information provided. Low total risk of bias seemed to be only valid for half of the included studies, aligning with low risk of bias across the remaining domains. A total of three studies were commercially sponsored [12, 15, 16]. Overall, the quality of the included studies was mixed, with only 62.5% of the studies at low risk of bias for attrition. All of the studies included were double-blinded, randomized controlled trials as per our inclusion criteria; however, only five studies delivered sufficient information on the methods implied to grant blinding of personnel and patients. Similarly, outcome reporting bias and random sequence generation have shown to be inconsistent, resulting in a possible influence on the significance of our results. Figures 2 and 3 demonstrate the results of the risk bias assessment.

### Efficacy of BTX Injections

**Fig. 4:**
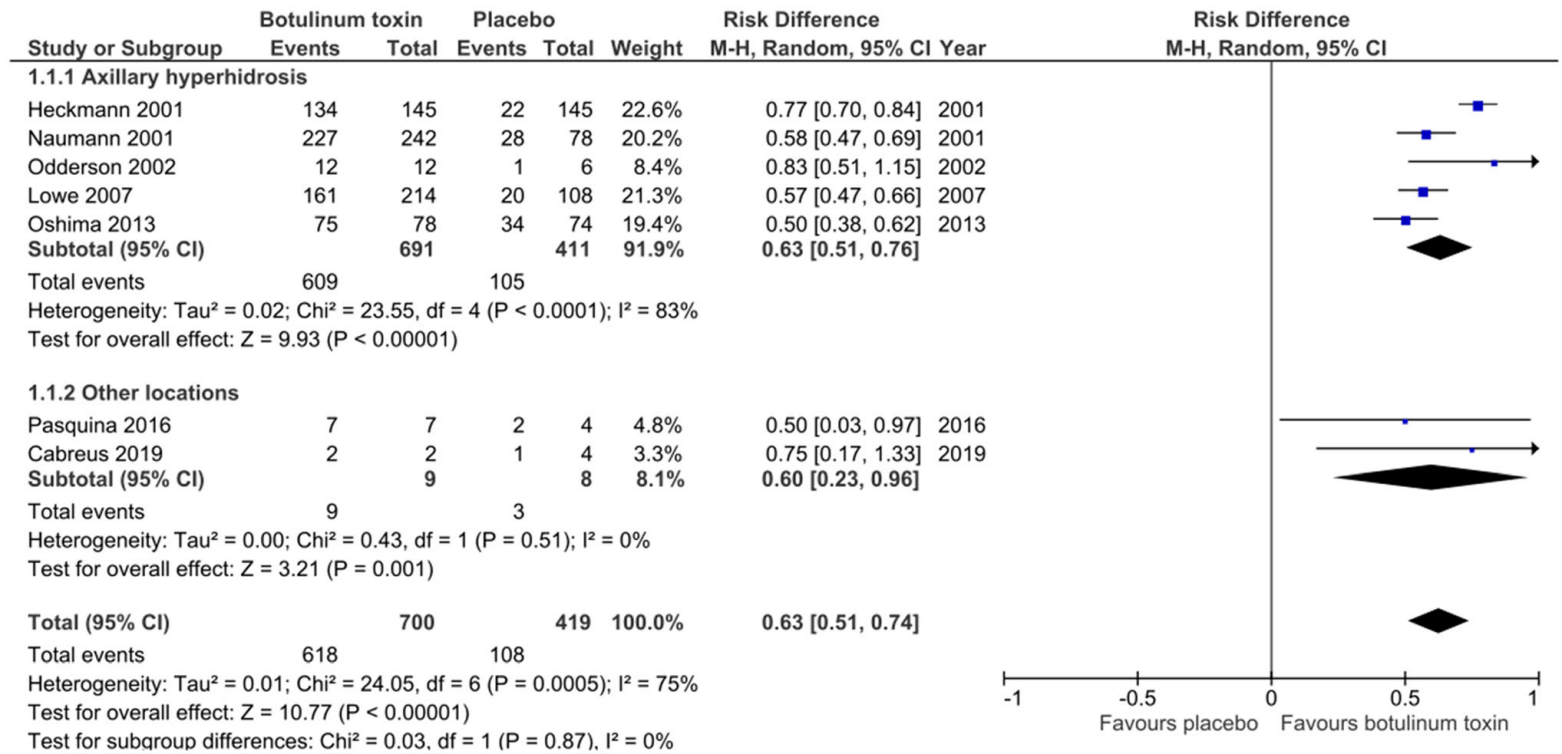
Comparison of botulinum toxin vs. placebo: gravimetric sweat reduction of ≥ 50% from baseline at weeks 2–6

**Fig. 5:**
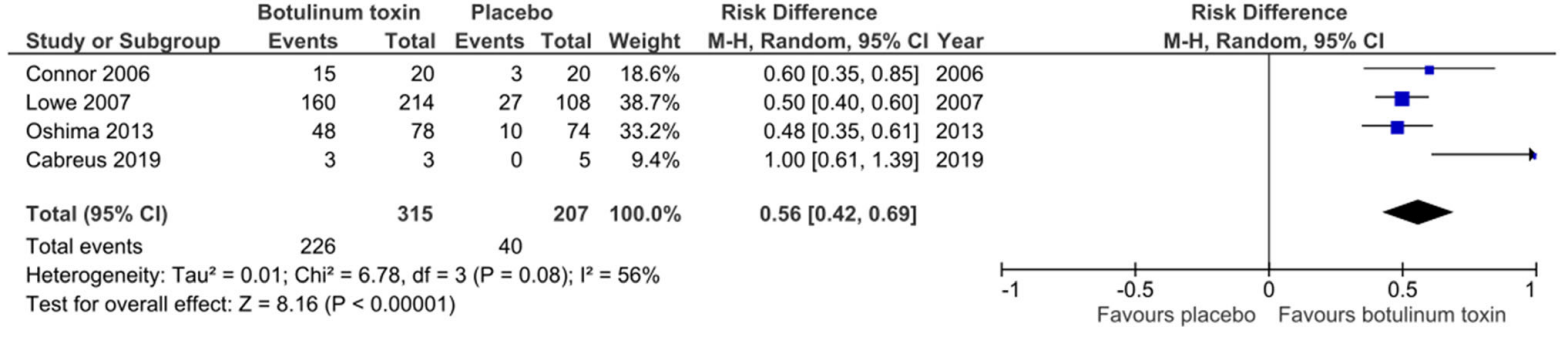
Comparison of botulinum toxin vs. placebo: reduction of ≥ 2 points in Hyperhidrosis Disease Severity Scale at weeks 2–8

**Fig. 6:**
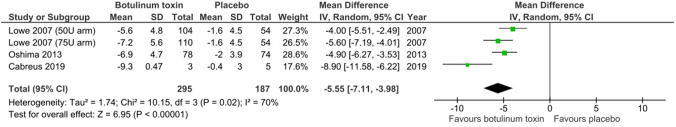
Comparison of botulinum toxin vs. placebo: mean change from baseline in Dermatology Life Quality Index score at weeks 2–8

Eight studies describing the effect of BTX injections for the treatment of focal hyperhidrosis were included. The serotype used in given trials differed, with six studies using BTX A and two studies using BTX B with a dose range of 50–200 units and 2.250–20.000 units, respectively. Placebo treatment using 0.9% sodium chloride solution was compared to BTX injections for axillary hyperhidrosis in six studies [12, 14–18], for craniofacial [13] and lower limb [19] hyperhidrosis in one study, respectively. We were able to evaluate the following outcome parameters for the comparative meta-analysis with placebo: > 50% sweat reduction from baseline at weeks 2–6 in the gravimetric analysis expressed as risk difference (RD) for axillary hyperhidrosis (RD: 0.63, 95% confidence interval (CI) [0.51, 0.76], *Z* = 9.93, *P* < 0.0001, *I*^2^ = 83%, five studies) and for craniofacial and lower limb hyperhidrosis (RD: 0.60, 95% CI [0.23, 0.96], *Z* = 3.21, *P* < 0.001, *I*^2^ = 0%, two studies) (Fig. 4); reduction of ≥ 2 points on the Hyperhidrosis Disease Severity Scale at week 2–8 expressed as RD (RD: 0.56, 95% CI [0.42, 0.69], *Z* = 8.16, *P* < 0.00001, *I*^2^ = 56%, four studies) (Fig. 5); and mean change in Dermatology Life Quality Index at weeks 2–8 from baseline expressed as mean difference (MD) (MD: − 5.55, 95% CI [ − 7.11, − 3.98], *Z* = 6.95, *P* < 0.00001, *I*^2^ = 70%, four studies) (Fig. 6).

## Discussion

All in all, our results hint at a pronounced and significant effect in favor of BTX injections over the course of eight weeks with clinically relevant sweat reduction and improvements in subjective quality-of-life. However, there is a considerable lack of evidence regarding given association in the mid- to long run and hyperhidrosis sites that were not listed in our included trials.

Hyperhidrosis presents a common condition in clinical practice and despite many treatment options available, the results frequently remain unsatisfactory. The condition assumes several facets of daily life with almost 40% of patients describing physical distress [20]. Accordingly, several aspects determining the quality-of-life such as psychological, professional and emotional well-being may be significantly impaired. In addition to the added multi-generational burden that comes with the diagnosis of the far-reaching disease [21], comorbidities, e.g., psoriasis and various other dermatologic conditions may develop consecutively.

Until today, BTX treatment remains one of the most commonly performed noninvasive aesthetic procedures and the neurotoxin has been standing firm as an effective and predictable agent. An avalanche of indications that extend beyond the known profound cosmetic benefits can be derived by BTX use [22]. With its high anhidrotic efficacy, adequate safety profile and favorable impact on patients’ quality of life, BTX has also quickly proven to be an efficient multidisciplinary therapeutic option in the treatment of hyperhidrosis. Based on the results of this meta-analysis, it could be inferred that BTX injections seem to be superior over the use of placebo.

Our study results showed promising implications for patients seeking medical help in practice for the treatment of hyperhidrosis. Based on mostly moderate quality evidence from eight randomized controlled trials, BTX injections derived superior gravimetric sweat reduction rates of ≥ 50% from baseline at week 2–6. For patients with debilitating hyperhidrosis and psychosocial distress, likely refractory to first line treatment, a significant sweat reduction may certainly represent a profound effect.

Similarly, the reduction of ≥ 2 points on the Hyperhidrosis Disease Severity Scale and the mean score reduction of the Dermatology Life Quality Index from baseline within 2–8 weeks showed to be superior. Even though these effects were derived from trials with limited patient cohorts and moderate quality evidence, the differences in severity scores indicate that BTX could significantly improve overall disease severity and quality-of-life.

Neither BTX effects nor quality-of-life or safety outcomes were available for long-term evaluation. Our results are accordingly limited to the first 8 weeks after initial injection. Therefore, the long-term determination of treatment effect would still be warranted in the future.

Current guidelines recommend BTX application after failure of topical agents [23]; however, study data suggest its superior efficacy in hyperhidrosis patients in comparison with first line topical treatments. Due to the lack of efficacy comparison with other treatment agents, conclusions cannot be drawn from our analysis to adjust the order of treatment modalities and the role of BTX injections. However, the confidence in the efficacy of the alternative treatment options is arguably no greater than that for BTX.

Despite its significant treatment effect and appropriate safety profile, common downsides need to be considered when administering BTX in the treatment of hyperhidrosis. Treatment costs are still high in comparison with readily available topical and systemic agents, especially when considering the necessity of multiple injections over the course of longer treatment periods. In our analysis, the lack of direct comparisons with other treatment modalities prevents the evaluation of cost effectiveness of the BTX treatment. Were it not for the significantly higher costs, it would probably be recommended earlier for hyperhidrosis care.

Besides, the pain induced by injections, particularly in sensitive areas such as the palms or axillae, can negatively impact patient comfort and tolerance, as well as long-term adherence [7]. Given the chronic character of the disease and the requirement for multiple injections during the course of the long-term treatment, transdermal BTX applications may be of interest in the future [24, 25]. Additionally, the minimal risk of anaphylactoid reactions and the risk of muscle atrophy with subsequent intramuscular fat deposition should always be born in mind and discussed with the patient prior to injection [26, 27]. Skilled injections and substantial knowledge of anatomical target sites are therefore of utmost importance for safe application. Studies included in our analysis generally depict that BTX has a substantial safety profile and the results from all included trials that evaluated adverse events agree with those assertions.

Nonetheless, given current guidelines on the treatment of hyperhidrosis, BTX therapy remains one of the most well-studied treatment options for focal hyperhidrosis and remains a solid treatment modality following unsuccessful initial topical treatment. [23]. Of note, a total of three BTX A products are cleared for the treatment of axillary hyperhidrosis, whereas the treatment of other focal sites has yet to be approved, rendering any such treatment currently off-label [28]. Further research on the efficacy of BTX injections is warranted in order to extend its approval and broader application for various focal sites affected by hyperhidrosis.

Regarding the limitations of this meta-analysis, a large-scale and adequately powered randomized controlled trial analyzing the effectiveness of BTX in comparison with established treatment modalities such as topical treatments, iontophoresis and surgical options would be warranted.

Additionally, further comparative studies with radiofrequency thermotherapy and fractional microneedle radiofrequency in hyperhidrosis may also be warranted. Rummaneethorn et al. showed in a prospective, randomized, split-side comparative study between BTX A injections and fractional microneedle radiofrequency in axillary hyperhidrosis a significantly superior reduction of mean HDSS scores and significantly better participant satisfaction scores for the BTX injections 12 weeks after the intervention [29]. However, given that thermotherapeutic options allow the maintenance of the skin’s integrity and reduction of patient discomfort and downtime, they may be a valid alternative to BTX injections [30].

## Conclusion

Our data suggest that BTX effectively yields superior results for patients with hyperhidrosis in terms of subjective and quantitative analysis compared to placebo. However, the limited quality of evidence needs to be considered.

Despite there being no gold standard in the treatment of hyperhidrosis, the current therapy scheme advocated recommends the use of BTX after the failure of conventional therapy. However, given its significant anhidrotic superiority over placebo injections, initial BTX treatment may be a good option in clinically severe cases in order to improve treatment response and to avoid unwarranted patient dissatisfaction. General downsides to consider in comparison with treatment alternatives would be discomfort as a result of injection-pain and substantially higher treatment costs.
